# Optimizing﻿ ethyl cellulose-ethanol delivery towards enabling ablation of cervical dysplasia

**DOI:** 10.1038/s41598-021-96223-9

**Published:** 2021-08-19

**Authors:** Jenna L. Mueller, Robert Morhard, Michael DeSoto, Erika Chelales, Jeffrey Yang, Corrine Nief, Brian Crouch, Jeffrey Everitt, Rebecca Previs, David Katz, Nimmi Ramanujam

**Affiliations:** 1 Department of Bioengineering, University of Maryland, 3102 A. James Clark Hall, 8278 Paint Branch Drive, College Park, MD 20742 USA; 2 Marlene and Stewart Greenebaum Cancer Center, University of Maryland School of Medicine, Baltimore, MD USA; 3 Department of Biomedical Engineering, Duke University, Durham, NC USA; 4 Department of Pathology, Duke University School of Medicine, Durham, NC USA; 5 Department of Obstetrics and Gynecology, Division of Gynecologic Oncology, Duke University Medical Center, Durham, NC USA; 6 Duke Global Health Institute, Duke University, Durham, NC USA; 7 Department of Pharmacology and Cancer Biology, Duke University, Durham, NC USA

**Keywords:** Targeted therapies, Gynaecological cancer, Cervical cancer, Cancer therapy

## Abstract

In low-income countries, up to 80% of women diagnosed with cervical dysplasia do not return for follow-up care, primarily due to treatment being inaccessible. Here, we describe development of a low-cost, portable treatment suitable for such settings. It is based on injection of ethyl cellulose (EC)-ethanol to ablate the transformation zone around the os, the site most impacted by dysplasia. EC is a polymer that sequesters the ethanol within a prescribed volume when injected into tissue, and this is modulated by the injected volume and delivery parameters (needle gauge, bevel orientation, insertion rate, depth, and infusion rate). Salient injection-based delivery parameters were varied in excised swine cervices. The resulting injection distribution volume was imaged with a wide-field fluorescence imaging device or computed tomography. A 27G needle and insertion rate of 10 mm/s achieved the desired insertion depth in tissue*.* Orienting the needle bevel towards the outer edge of the cervix and keeping infusion volumes ≤ 500 µL minimized leakage into off-target tissue. These results guided development of a custom hand-held injector, which was used to locate and ablate the upper quadrant of a swine cervix in vivo with no adverse events or changes in host temperature or heart rate. After 24 h, a distinct region of necrosis was detected that covered a majority (> 75%) of the upper quadrant of the cervix, indicating four injections could effectively cover the full cervix. The work here informs follow up large animal in vivo studies, e.g. in swine, to further assess safety and efficacy of EC-ethanol ablation in the cervix.

## Introduction

In high-income countries, cervical cancer rates have dropped significantly in the past fifty years, primarily due to development and implementation of population screening with the Papanicolaou (Pap) smear, and more recently with co-testing for human papilloma virus (HPV)^1^. HPV vaccination has the potential to further decrease cervical cancer rates; however, HPV vaccination rates vary widely within and between countries with limited coverage in low-income areas^2,3^. In low- and middle-income countries (LMICs), cervical cancer remains the second highest cause of cancer-related death in women^4^. Barriers to cervical cancer prevention include few trained providers, social stigma, lack of awareness, limited access to providers, and limited access to biomedical technologies needed to prevent, diagnose, and treat cervical dysplasia before it becomes cancer^5–7^.

A three-visit paradigm is commonly used in high-income settings to prevent cervical cancer. First, women are screened with Pap smear and HPV co-testing at 3–5 year intervals depending on age and risk for high-grade cervical dysplasia (CIN3)^8^. Based on screening results and clinical history, patients who are at high risk for CIN3 are referred for colposcopy-guided biopsy to confirm disease^8^. Patients with an elevated risk of CIN3 are treated with an excisional outpatient procedure called Loop Electrosurgical Excision Procedure (LEEP) or cold knife cone during which the lesion and the cervical transformation zone are excised for further pathologic evaluation^1^. LEEP removes a significant proportion of cervical tissue including much of the ectocervix. While this works wells in high-income settings where follow up diagnosis and treatment are abundant, it is of greater concern in settings where regular gynecological monitoring and treatment may not be available or practiced. For example, a number of studies have reported that LEEP increases risk for cervical shortening, cervical insufficiency, and spontaneous preterm labor^9–12^. Diagnosis and treatment of these sequelae can also be compromised in LMICs, and these concerns add motivation to implementation of a practical alternative to LEEP in such settings. Further, LEEP requires an experienced provider to perform the procedure, as well as constant electricity, specialized equipment, and resources to manage serious complications; these limit its use in LMICs^13^.

In light of the above, the World Health Organization (WHO) recommends adoption of alternative protocols in LMICs that employ accessible technologies to screen and treat cervical pre-cancer in a single visit with a primary care provider^14^. In this regard, significant technological advances have been made in developing more accessible HPV testing^15^ and low-cost colposcopy^16^. However, accessible screening and diagnosis alone will not ensure decreases in cervical cancer mortality if access to point-of-care treatment remains limited. In some LMICs, up to 80% of women who have been diagnosed with cervical dysplasia never receive treatment^17^. Consequently, the WHO recommends the use of ablative therapies, including cryotherapy and thermocoagulation, to treat cervical pre-cancer in LMICs because they cost less than excisional procedures, do not require removing tissue, therefore leaving the cervix intact, and can be performed by non-specialists in primary care settings^14^. Cryotherapy uses compressed carbon dioxide or nitrous oxide gas and a metal probe to freeze abnormal tissue. While cryotherapy is considered low-cost compared to excisional procedures, it requires high quality gas tanks, which are difficult to consistently procure and transport to rural communities in LMICs^18,19^. Thermocoagulation, an ablative method in which heat is used to destroy cervical lesions, is another approach gaining popularity because of ease-of-use^13^. Specifically, it uses electricity to heat a probe to 100 °C, which is brought into contact with a lesion for 20–60 s to cause necrosis in the region that is in contact with the probe^13^. While thermocoagulation is easy to administer, its reliance on electricity as a power source can limit its use in LMICs^13^. In practice, cryotherapy and thermocoagulation are based on bioheat transfer and consequently are limited with respect to the depth of penetration of the treated zone in tissue. CIN3 varies in extent from a mean depth of 1.35 mm up to a maximum of 4.8 mm^20^. Studies indicate carbon dioxide based cryotherapy achieved an average depth of necrosis of 3.4 mm^21^, and thermocoagulation achieved an average depth of 2.6 to 3.5 mm depending on duration and temperature^22^. Thus, bioheat transfer limits the range of geometries of target tissue that can be treated with cryotherapy or thermocoagulation. Further, neither therapy is effective in treating disease extending into the endocervical canal^13^. Thus, there is an unmet need for an accessible, robust therapeutic for LMICs that could be expanded to treat disease in the endocervical canal and for treatment of early carcinomas.

Ethanol ablation, which entails injecting ethanol into a lesion to cause necrosis through protein denaturation and cytoplasmic dehydration^23^, is a potential alternative to cryotherapy and thermocoagulation that could be accessible in LMICs. This procedure only requires the use of medical grade ethanol and a sterile syringe and needle, which are low-cost, portable, and widely available in LMICs. In high-income countries, ethanol ablation has previously been used to treat inoperable hepatocellular carcinomas^24^, parathyroid tumors^25^, and pancreatic tumors^26^. While conventional ethanol ablation has a long history of clinical use, direct injection of ethanol into tissue can lead to non-uniform distribution and low ablation efficacy in the region of interest. For example, fluid leakage during ethanol ablation of hepatocellular carcinomas is well-documented and can cause vascular and bile duct injuries^27^ and necrosis of healthy peripheral tissue^28^. Fluid leakage can occur through: (1) retrograde flow parallel to the needle and up toward and to the tissue surface (i.e. backflow), particularly in more superficial injections; or (2) extended flow away from the needle tip into less dense tissue or body cavities adjacent to the lesion (via formation of cracks within the tissue into which the injectate flows). In practice, ethanol ablation of hepatocellular carcinoma often requires multiple sessions to achieve the same efficacy as other ablative methods^29^. To reduce leakage and increase ethanol retention in injected lesions, we added the polymer ethyl cellulose (EC) to the injectate^30^. EC was selected because it is low-cost, generally regarded as safe by the Food and Drug Administration^31^, readily dissolved in ethanol, and increased the viscosity of the injectate, which reduced backflow^32,33^. Further, the ethanol-EC mixture formed a highly viscous gel when contacting the aqueous environment within tissue, which increased ethanol retention at the injection site^30^.

Initial in vivo studies to evaluate therapeutic efficacy of EC-ethanol were carried out in a hamster-cheek pouch model of oral squamous cell carcinoma^30^. Tumors were injected with 50 µL (25% of the tumor volume) of either pure ethanol or 3% EC-ethanol, at infusion rates ranging from 0.1 mL/h up to 100 mL/h (a common rate for manual injections). EC-ethanol injected at an infusion rate of 10 mL/h achieved complete regression of all tumors 7 days after injection; this contrasted with 0% tumor regression for an equal volume of pure ethanol injected at 100 mL/h^30^. Similarly, when applied to treat a murine breast tumor model, EC-ethanol yielded decreased localized adverse events, a fivefold increase in tumor necrotic volume, and longer survival rates compared to the same volume of pure ethanol, further indicating that EC-ethanol is safer and more effective than ethanol alone^34^. Additional in vitro studies were conducted in excised swine liver tissue to begin to understand how salient injection parameters impacted injected fluid distribution. Tissue was injected with EC-ethanol mixed with fluorescein (to enable visualization of injectate distribution) using a range of injection protocols in which the concentration of EC, infusion rate, and infusion volume were systematically varied^35^. Fluid leakage remained low for 6% EC-ethanol compared to 3% EC-ethanol and pure ethanol, regardless of infusion rate. Additionally, infusion pressure scaled with infusion volume, and there was a critical infusion pressure beyond which crack formation occurred, leading to fluid leakage.

Our initial studies, summarized above, show promise for use of ethanol ablation in efficacious treatment of cervical dysplasia in LMICs. There are critical next steps in understanding the biophysical and pathophysiological mechanisms of this methodology, and in implementing it in a relevant large animal setting. The present study addressed these. We focused further upon the best EC-ethanol formulation and delivery parameters to achieve optimal distribution in the cervix, considering the histology of cervical dysplasia, and conducting studies in swine cervices, which are comparable in size to human cervices^36^. We also began development and validation of a hand-held automated injection device for in vivo application. Ex vivo swine cervix experiments were conducted in establishing design details for optimal injections. We then performed an initial cervical ablation experiment in vivo with the new device in a living pig to demonstrate feasibility. Thus, the novelty of this study includes the first application of EC-ethanol ablation to the cervix in a large animal model, ex vivo and in vivo. The latter included development of an injection device to deliver EC-ethanol to the porcine cervix in vivo. Overall, in this study we: (1) performed injections in the transformation zone around the os, which is the site most impacted by dysplasia; (2) optimized key injection delivery parameters to achieve requisite injection cloud volume and depth; (3) described development and feasibility of a novel injector to deliver EC-ethanol to a swine cervix in vivo; and (4) conducted a pilot in vivo cervical ablation experiment with the novel injector. We found that a 27G needle and insertion rate of 10 mm/s maximized insertion depth into tissue. Orienting the needle bevel towards the outer edge of the cervix minimized off-target leakage. Injected volumes ≤ 500 µL injected at 10 mL/h led to maximal fractional retained volumes within the tissue. The custom injector automated needle insertion and delivery (27G needle, 10 mm/s insertion rate, 10 mL/h infusion rate, 500 µL infusion volume based on ex vivo experiments), and it successfully enabled delivery of EC-ethanol to cervical tissue in vivo, achieving a distinct volume of necrosis that covered a majority (> 75%) of the upper quadrant of the cervix after 24 h. The study here lays the groundwork for follow up, thorough investigation of ethanol-EC injection in a large animal model in vivo, prerequisite to evaluation in humans.

## Results

### Optimizing needle insertion and orientation to minimize backflow

**Figure 1:**
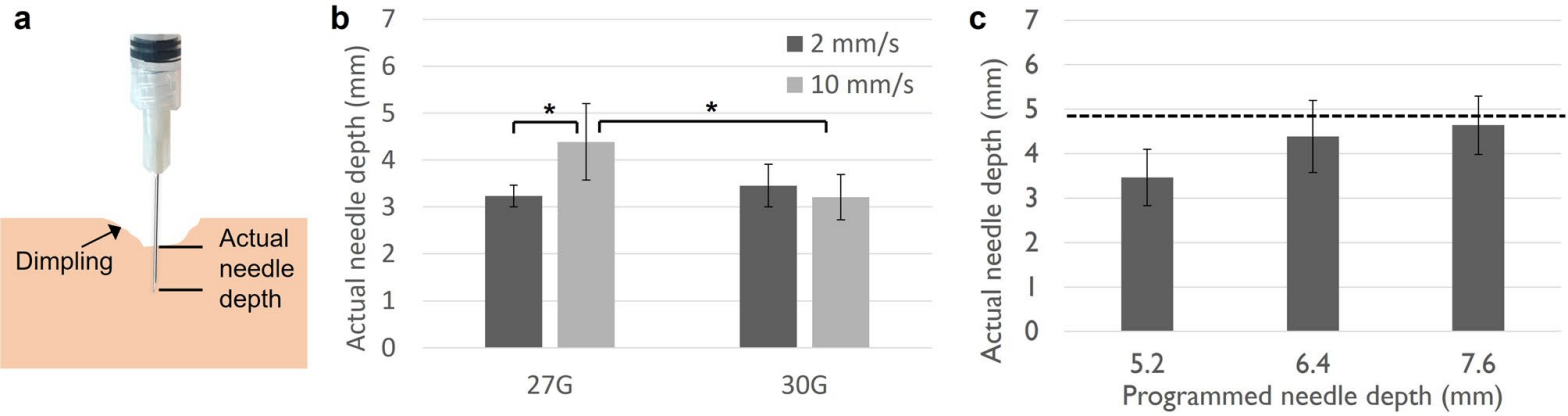
Optimizing needle insertion to minimize dimpling. (**a**) Schematic illustrating that dimpling leads to lower actual needle depths compared to the programmed needle depth. (**b**) Relationship between needle gauge, needle insertion rate, and actual needle depth in the swine cervix for a programmed needle depth of 6.4 mm. A 27-gauge needle and insertion rate of 10 mm/s achieved the highest actual needle depth. (**c**) Relationship between programmed needle depth and actual needle depth in a swine cervix for a 27-gauge needle and an insertion rate of 10 mm/s. A programmed needle depth of 7.6 mm achieved an actual needle depth close to a target of 4.8 mm (indicated by dashed line). All groups had a sample size of n = 4 to 5. *p < 0.05.

Different needle gauges and insertion rates were investigated in ex vivo swine cervices to minimize dimpling, which occurs when the tissue is soft or resistant to needle insertion (Fig. 1a). Both 27 and 30-gauge (G) needles were compared, each at insertion rates of 2 and 10 mm/s, to determine their impact on dimpling, which was assessed by calculating the difference between programmed and actual needle depths. The needle diameters, 27G and 30G needles were selected because their small diameters decrease pain upon insertion^37^, while larger needle diameters can lead to more tissue damage and backflow to the tissue surface^38^. Needle insertion rates were selected based on previous experiments, which indicated that reduced dimpling was observed with rates ≥ 2 mm/s^35^. A 27G needle, inserted at a rate of 10 mm/s to a programmed needle depth of 6.4 mm, yielded the highest actual insertion depth and minimized dimpling (Fig. 1b). Next, the programmed needle depth was varied for a 27G needle diameter and a 10 mm/s insertion rate. A programmed needle depth of 7.6 mm achieved a penetration depth of approximately 4.8 mm, which is the depth needed to treat high-grade dysplasia (Fig. 1c).

### Optimal orientation of the bevel

**Figure 2:**
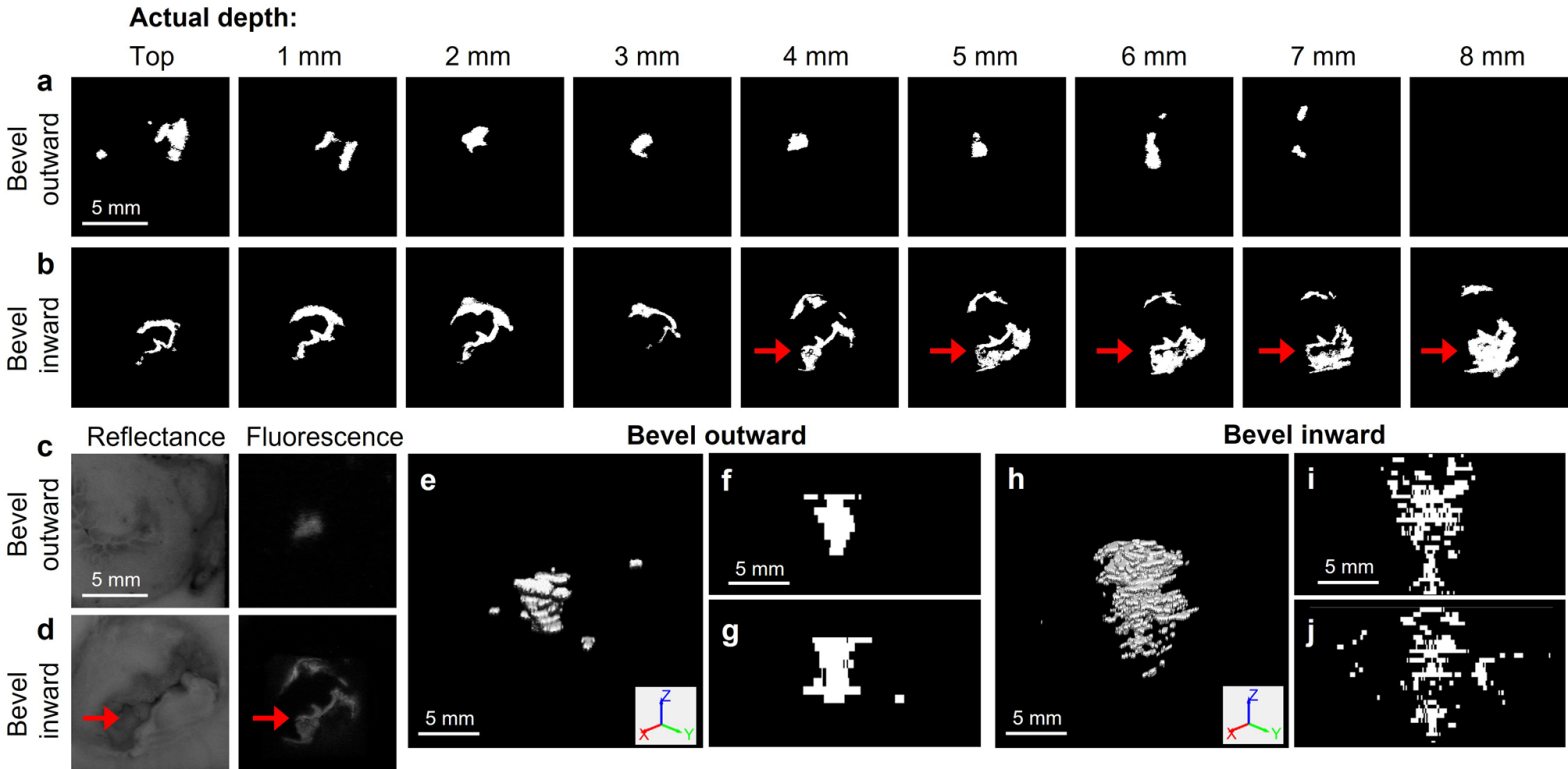
Representative thresholded fluorescein images from an ex vivo swine injection as a function of actual needle depth (27-gauge needle, 7.6 mm insertion depth, 10 mm/s insertion rate, 6% EC-ethanol, 10 mL/h infusion rate, and 200 µL infusion volume) in which the needle bevel was facing (**a**) outward to the outer edge of the cervix and (**b**) inward towards the os. Five minutes post injection, the tissue was flash frozen and subsequently sectioned with a cryostat and imaged at various depths (starting from the top of the sample and moving downward into the sample). Red arrows indicate leakage into the os, which was localized by comparing reflectance images to the corresponding fluorescence images. Representative reflectance and fluorescence images from a 4 mm depth when the bevel was facing (**c**) outward and (**d**) inward. (**e**) 3D rendering of reconstructed volume of the injection in which the needle bevel was facing outward and slice through the center of the (**f**) XZ and (**g**) YZ planes. (**h**) 3D rendering of reconstructed volume of the injection in which the needle bevel was facing inward and slice through the center of the (**i**) XZ and (**j**) YZ planes. Scale bars are 5 mm.

Next, effects of orientation of the needle bevel were investigated. Specifically, the bevel was oriented either outward towards the other edge of the cervix or inward towards the os. Fluorescein was mixed with 6% ethyl cellulose (EC)-ethanol and injected into ex vivo swine cervices using a 27G needle, 10 mm/s insertion rate, and 7.6 mm programmed depth. Cervices were flash frozen, sectioned, and imaged with a wide-field fluorescence imaging device. Images of representative injections where the needle was oriented outward and inward are shown at different depths in Fig. 2. As seen, a clear depot forms around the needle tip when the bevel is oriented outwards. When the bevel faces inwards, distribution within the endocervical canal (which is indicated by the red arrows) is visible.

**Figure 3:**
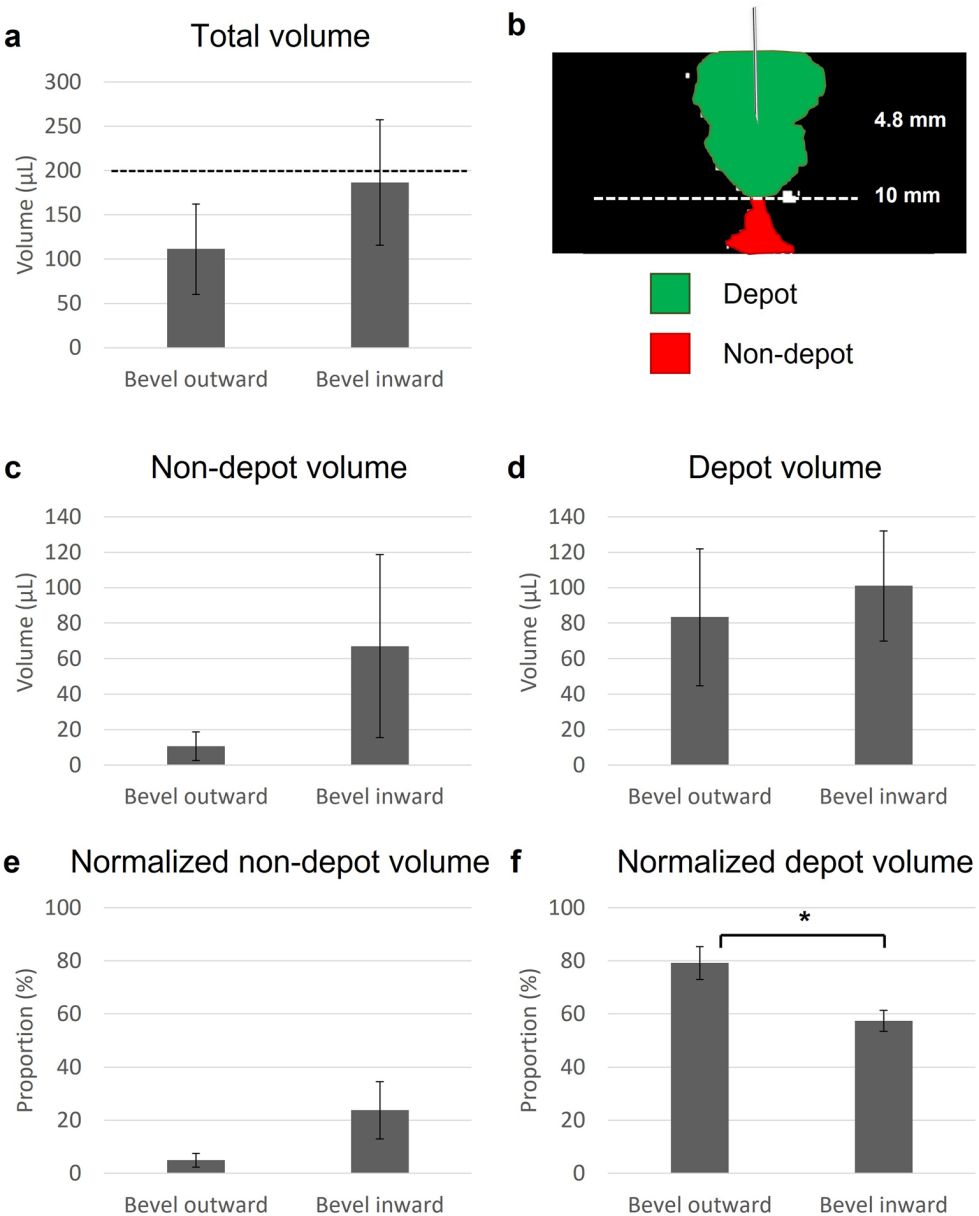
Variation across ex vivo swine injections (7.6 mm insertion depth, 10 mm/s insertion rate, 6% EC-ethanol, 10 mL/h infusion rate, 200 µL infusion volume). For half of the injections, the needle bevel was facing outward (towards the outer edge of the cervix), and for the other half, the needle bevel was facing inward (towards the os). To assess the amount of fluid localized at the needle tip, first (**a**) total distribution volume was calculated. The programmed infusion volume of 200 µL is indicated by the dotted black line. (**b**) Then, fluid was segmented into (**c**) depot volume, which was defined as the largest region around the needle tip in the top 10 mm of the tissue, and (**d**) non-depot volume, which was defined as the fluid below 10 mm into the tissue. (**e**) The normalized non-depot volume (non-depot volume/total distribution volume) quantified to proportion of fluid outside of the depot (in cracks). (**f**) The normalized depot volume (depot volume/total distribution volume) quantified the proportion of fluid within the depot (around the needle). As seen, normalized depot volume was significantly higher when the bevel was facing outward, likely because there was less leakage into the os. All groups had a sample size of n = 4 to 5. *p < 0.05.

Several parameters were calculated to quantify the amount of fluid localized about the needle tip (Fig. 3). First, total distribution volume was calculated. Then, fluid was segmented into: depot volume, which was defined as the radially symmetric region around the needle tip; and non-depot volume, which was defined as the fluid outside of the depot in cracks within the tissue. The transition depth between depot and non-depot volume was set at 10 mm, which was the approximate lower bound for the spherical region. Normalized depot volume quantified the proportion of fluid within the depot relative to the total distribution volume. Total volume and non-depot volume were higher when the bevel was facing inward. Depot volume was comparable across the two needle orientations, indicating that more fluid is leaking outside of the depot when the bevel faces inward. Additionally, normalized depot volume was significantly higher when the bevel was facing outward. This further indicates that orienting the bevel outwards minimizes off-target leakage and results in a more homogenous distribution volume.

### Injector testing

**Figure 4:**
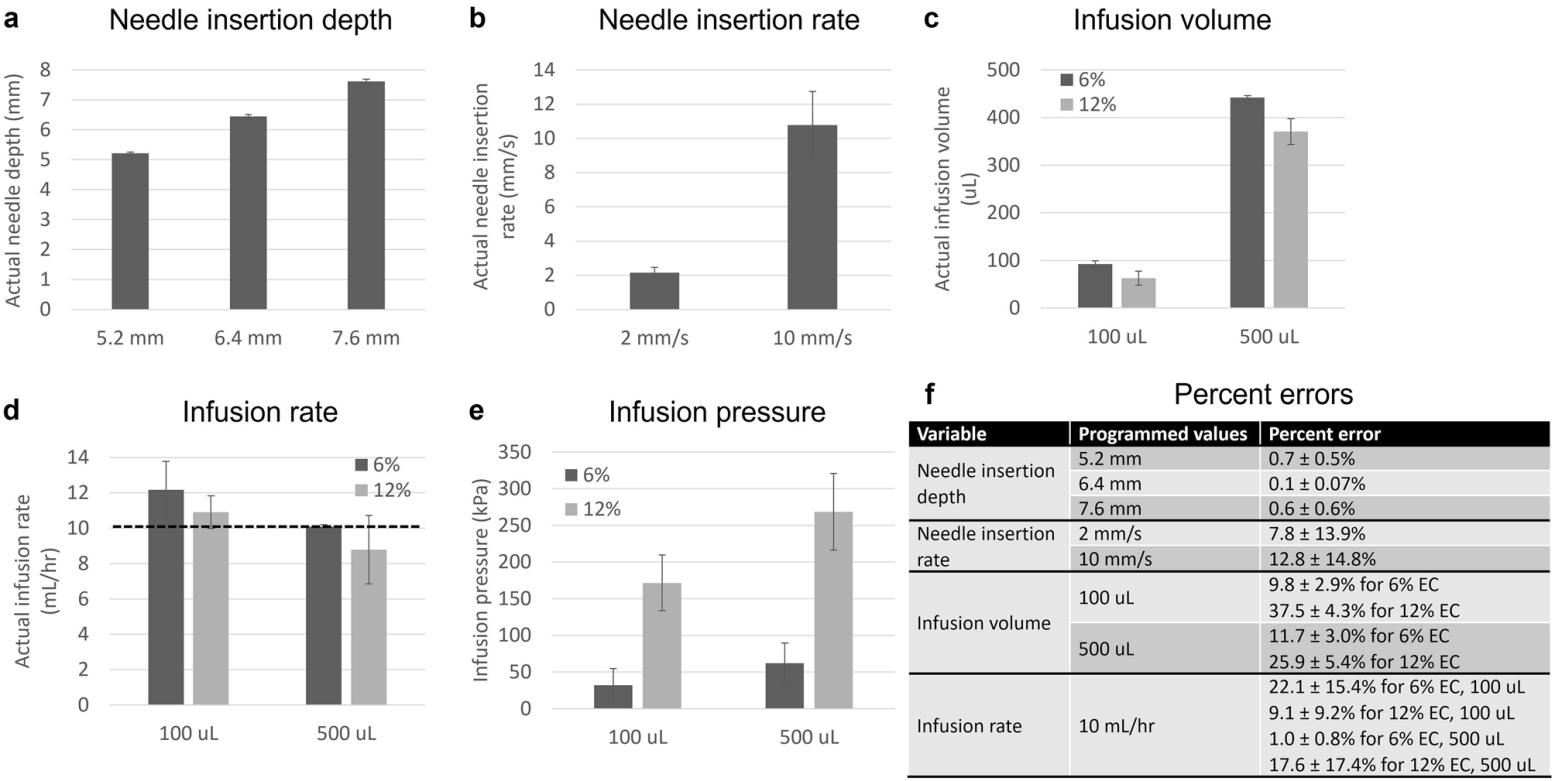
Injector benchtop validation. (**a**) Needle insertion depth was measured with calipers for programmed needle depths of 5.2, 6.4, and 7.6 mm. (**b**) The needle insertion rate was measured by calculating the needle movement over time for programmed needle depths of 2 and 10 mm/s. (**c**) A programmed infusion volume of either 100 or 500 µL of 6% or 12% EC-ethanol was injected into a container from which mass (and the corresponding actual infusion volume) could be measured. (**d**) The infusion rate was verified by measuring the time required to deliver programmed volumes of 100 and 500 µL into a container. (**e**) Infusion pressure was also monitored during injections with the manometer. Percent errors for all benchtop testing are summarized in (**f**). All groups had a sample size of n = 5.

A series of bench tests was performed to assess the performance of the hand-held injector (Fig. 4). These tests were performed in two groups: 1) the accuracy of needle insertion rate and depth were assessed by programming different values and measuring the needle movement in air (i.e. no injections were performed); and 2) the accuracy of specifiying infusion volume and rate was assessed by loading in different concentrations of EC-ethanol and measuring the mass injected into an empty container. Specifically, the needle insertion rate was measured by observing the needle movement over time for programmed needle rates of 2 and 10 mm/s. The insertion rate was within 8% and 13% of the programmed rate for 2 and 10 mm/s, respectively. Next, insertion rate was held constant at 10 mm/s, and programmed needle insertion depth was varied from 5.2 to 7.6 mm. The actual needle depth was measured with calipers. For all depths, the measured needle depth was within 1% of the programmed depth.

In the second set of experiments, needle insertion rate and depth were held constant at 10 mm/s and 7.6 mm, respectively. Two different percentages of EC in ethanol (6% and 12%) and infusion volumes (100 µL and 500 µL) were injected into empty containers at 10 mL/h. These values were selected because parallel studies have indicated that 500 µL of 12% EC-ethanol provides a greater distribution and depot volume compared compared to 100 µL of 6%-EC ethanol^39^. This is significant in that fewer injections would be required to cover the cervix. A volume of either 100 or 500 µL delivered at 10 mL/h was set through the LabVIEW interface, and 6% and 12% EC-ethanol were injected into an empty container from which the mass could be easily measured. For 6% EC-ethanol, the infusion volume was within 10% and 12% of programmed volume for 100 µL and 500 µL, respectively. For 12% EC-ethanol, the infusion volume was within 38% and 26% for 100 µL and 500 µL, respectively. The actual infusion volumes were smaller than programmed infusion volumes, particularly for the higher concentration of EC. An infusion rate of 10 mL/h was verified by calculating the time required to deliver programmed volumes of 100 and 500 µL. For 6% EC-ethanol, the measured infusion rate was within 23% and 2% of programmed rate for infusion volumes 100 µL and 500 µL, respectively. For 12% EC-ethanol, the measured infusion rate was within 10% and 18% of programmed rate for infusion volumes 100 µL and 500 µL, respectively. Lastly, infusion pressure was monitored during injections with the manometer. Increasing the volume from 100 to 500 µL increased infusion pressure by a factor of 1.9X for 6% EC-ethanol (from 33.0 ± 2.5 to 62.6 ± 0.8 kPa) and by a factor of 1.6X for 12% EC-ethanol (from 172.6 ± 22.5 to 277.8 ± 11.1 kPa). Increasing the EC concentration from 6 to 12% increased infusion pressure by a factor of 5.2X for 100 µL (from 33.0 ± 2.5 to 172.6 ± 22.5 kPa) and 4.4X for 500 µL (from 62.6 ± 0.8 to 277.8 ± 11.1 kPa). Taken together, the injector enabled programmed needle insertion with errors ≤ 13% and programmed infusion of 6% EC-ethanol with errors ≤ 22%. Infusion of 12% EC-ethanol led to larger errors (up to 38%) in infusion volume because the higher viscosity solutions required more force and did not flow as easily, which led to a smaller actual infusion volume than the programmed value.

### Determining the maximum infusion volume for swine injections

**Figure 5:**
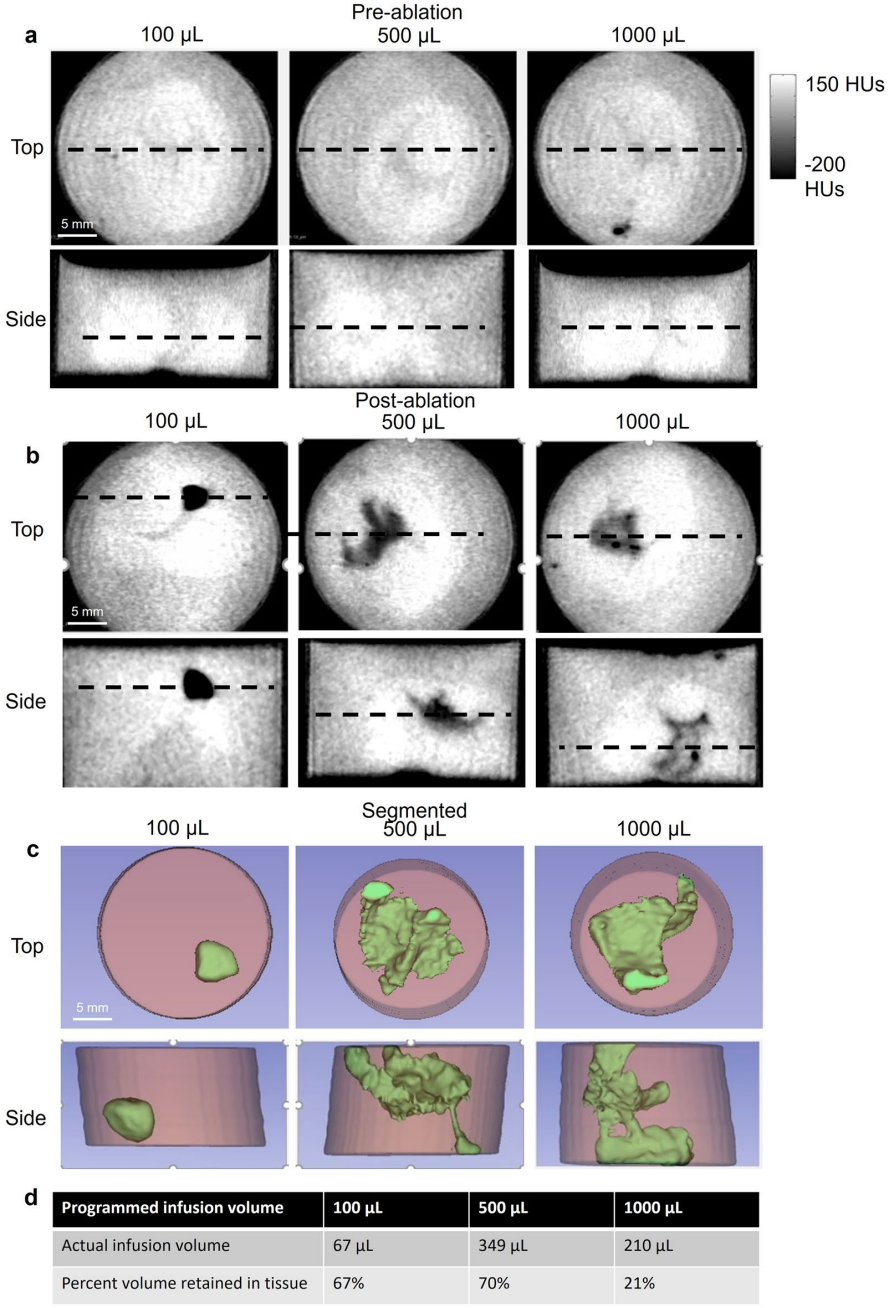
CT images 12% EC-ethanol in the ex vivo swine cervix (manual needle insertion, ~ 6.5 mm insertion depth, 12% EC-ethanol, 10 mL/h infusion rate, 100 µL, 500 µL, or 1000 µL infusion volumes). (**a**) Pre-ablation images obtained before the injection (baseline images shown from the top and side of the sample). Black dotted lines represent the plane of the corresponding image for each sample. (**b**) Post-ablation images were obtained approximately 5 min post injection. For each 2D image, the top and side view corresponding to the largest distribution are shown. Ethanol is less radiodense than cervix tissue and appears black in the image. (**c**) Segmentation of distribution volumes. A threshold of 0 HUs was applied to segment the ethanol region. Projections of top and sideviews of the cervix are shown where ethanol is false colored green. Scale bars are 5 mm. (**d**) Programmed infusion volumes of 100 µL, 500 µL, or 1000 µL infusion volumes led to distribution volumes of 67, 349, and 210 µL, respectively. The corresponding percentage volume retained within the tissue was 67%, 70%, and 21%, respectively, for the three infusion volumes.

To determine the maximum infusion volume that can be used without increasing fluid leakage, CT imaging was used to quantify ethanol distribution for different infusion volumes in intact ex vivo swine cervices (Fig. 5). Ethanol is less radiodense than cervical tissue and appears black in the image, which can be easily thresholded to calculate the distribution as well as corresponding percent retained within tissue. 12% EC-ethanol injected at 10 mL/h was used to deliver 100, 500, and 1000 µL into a swine cervix (manual needle insertion, ~ 6.5 mm insertion depth). Pre-ablation images were obtained before the injection, and post-ablation images were obtained approximately 5 min after the injection. (Fig. 5). Programmed infusion volumes of 100, 500, and 1000 µL infusion volumes led to 67%, 70%, and 21% of the initial volume retained in the tissue, respectively. These results indicate that the percent volume retained within the tissue stays relatively constant for up to 500 µL after which the percent volume drops significantly.

### Evaluating feasibility of EC-ethanol injection in an in vivo swine cervix model

**Figure 6:**
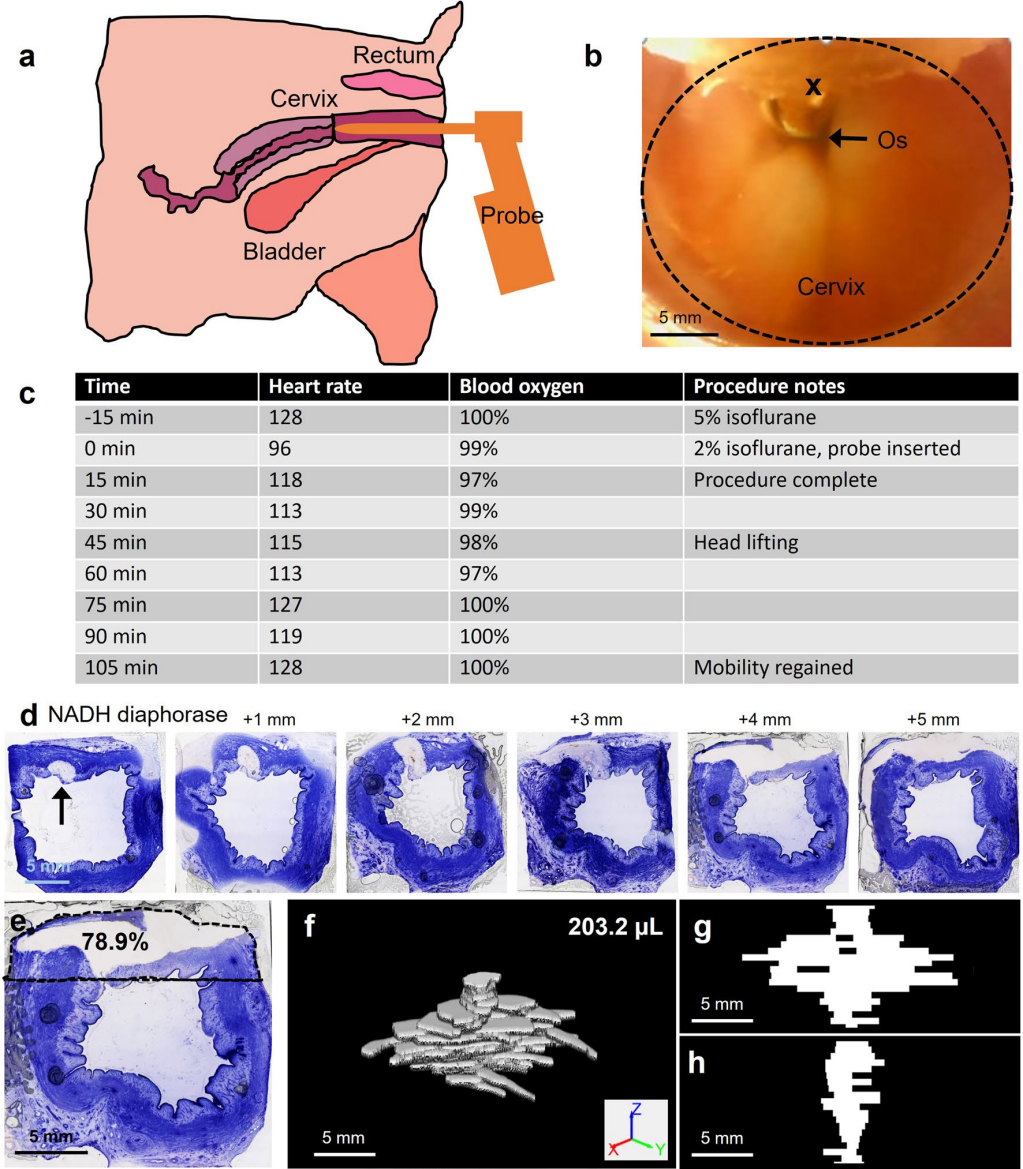
Feasibility of EC-ethanol injection in an in vivo swine cervix model (7.6 mm insertion depth, 10 mm/s insertion rate, 12% EC-ethanol, 10 mL/h infusion rate, 500 µL infusion volume). (**a**) Orientation of how our prototype injector was inserted into the vaginal canal and used to find (**b**) the cervical os. The cervix and injection location are indicated by a dashed black circle and black x respectively. (**c**) Heart rate and blood oxygen saturation were monitored throughout the procedure. Mobility was regained 90 min after the procedure was completed. (**d**) After 24 h the cervix was excised. Necrosis, seen as the region that did not stain blue with NADH diaphorase (and indicated by a black arrow in first image), could be clearly seen in the excised cervix. (**e**) At a 4 mm depth, the necrotic region covered 78.9% of the upper quadrant of the cervix (outlined by a black dotted line). (**f**) 3D rendering of reconstructed necrotic volume and slice through the center of the (**g**) XZ and (**h**) YZ planes. The volume of necrosis was 203.2 µL. Scale bars are 5 mm.

The findings from ex vivo tissue studies were used to select initial injection and delivery parameters in the in vivo feasibility study. The hand-held injector was used to program these variables and control delivery of EC-ethanol into the swine cervix in vivo. A schematic of the female anatomy is shown in Fig. 6a. First, the camera and LED light source incorporated into the injector were used to find the cervical os in vivo (Fig. 6b). Then the needle was inserted at 10 mm/s insertion rate to a depth of 7.6 mm (programmed values were based on results from Fig. 1), and 12% EC-ethanol was injected at 10 mL/h infusion rate to a volume of 500 µL (programmed values were based on results from Fig. 5). Vitals, including heart rate and blood oxygen, were recorded throughout the procedure (Fig. 6c). The swine gained mobility within 90 min of the procedure, and no respiratory distress was noted. After 24 h, the cervix was excised, flash frozen, sectioned every 500 µm, and stained with NADH diaphorase, which stains viable regions blue and does not stain necrotic regions. As seen in Fig. 6d, the necrotic region can be clearly seen at the top of the image. At a 4 mm depth, the necrotic region covered 78.9% of the upper quadrant of the cervix (Fig. 6e). Necrotic regions were outlined, reconstructed into a 3D volume with various views shown in Fig. 6f-h, and quantified in image processing software. At a 4 mm depth, the necrotic region covered 78.9% of the upper quadrant of the cervix, and the volume of necrosis induced by a 500 µL infusion volume was 203.2 µL.

## Discussion

EC-ethanol injection has the potential to be an alternative to current therapies to treat cervical pre-cancer in LMICs, since it is low-cost, portable, and does not require reliable access to electricity. Our initial studies demonstrated efficacy in treating oral squamous cell carcinomas in a hamster cheek pouch model^30^, and examined the relationship between several injection parameters and the final distribution volume in excised swine liver tissue^35^ and a murine breast tumor model^34^. An essential next step in development of this technique was to consider how to successfully apply EC-ethanol to the cervix, which requires accounting for the unique geometry of the cervix and of the location where dysplasia develops (the transformation zone). The results from the study here elucidated key considerations for delivery of EC-ethanol into the cervix, injector design, and needle-based drug delivery.

In safely and effectively treating cervical dysplasia by EC-ethanol ablation, it is necessary to optimize EC-ethanol delivery to the cervix by minimizing fluid leakage, which can occur through two primary pathways: (1) retrograde flow up the needle pathway (backflow) leading to pooling at the surface of the cervix; or (2) crack formation leading to fluid accumulation inside the endocervical canal. Backflow can be decreased by maximizing the contact area between the needle and surrounding tissue, creating a stronger seal. Specifically, dimpling (tissue deformation around the needle tip) leads to less contact area between the needle and tissue, which can result in more backflow^40^. Previously we found that a 27G needle inserted at 2 and 10 mm/s decreased dimpling in the excised swine liver^35^. Here we found that a 27G needle inserted at 10 mm/s led to more precise programmed depths and decreased dimpling in excised swine cervices. Higher insertion rates were needed when injecting swine cervical tissue likely because swine cervical tissue is softer and more likely to deform than swine liver tissue; thus, selecting actuators capable of inserting the needle at 10 mm/s was a key design consideration for the hand-held injector.

Another factor influencing fluid leakage was bevel orientation. Off-target leakage into the endocervical canal was minimal when the needle bevel was aimed laterally, away from the os towards the outer edge of the cervix. In previous experiments conducted in transparent tissue surrogates, we found that the injection depot volume was largest in the direction that the needle bevel was facing, because the flow of fluid is not perfectly symmetrical coming out of the needle bevel^30^. When the needle bevel was aiming medially inward, it was more likely that fluid was pushed into the endocervical canal. Thus, aiming the needle bevel laterally, away from the os, led to less off-target leakage and a more controlled depot volume around the tip of the needle.

Insights from our ex vivo experiments guided design of a hand-held injector, which was used to site a swine cervix in vivo, inject and control delivery of EC-ethanol. A camera and LED were incorporated into the tip of the injector body because the geometry of the swine vaginal canal does not allow for easy visualization of the cervix, and speculums are not regularly used in swine. While a camera and LED were necessary components to visualize the swine cervix in vivo, they may not be needed in an injector designed for the human cervix. Speculums are used during excisional and ablative procedures in order to help visualize the cervix and protect the vaginal walls. This provides precedent to use a speculum during EC-ethanol ablation in humans. Prior to gynecological excisional treatments, a needle attached to a needle extender is often used to inject a local anesthetic such as lidocaine into the submucosa. Here, the needle is carefully inserted through the speculum and into the cervix by the provider, guided by the naked eye. This established clinical approach could readily be modified to configure EC-ethanol delivery through the speculum into the cervix by adding actuators to the injector to control needle insertion rate and depth, and also EC-ethanol infusion rate and volume. Lastly, the number and location of injections in the human cervix will need to be considered. In our pilot in vivo experiment, one injection was performed in the 12 o’clock position, halfway between the cervical os and the vaginal wall. The resulting necrotic region covered a majority (> 75%) of the upper quadrant of the cervix at a depth of 4 mm (which corresponded approximately to the location of the tip of the needle). This initial result suggests that 4 equally spaced injections around the external os would lead to adequate coverage across the entire swine cervix, which is similar in size to the human cervix^36^.

The methodology presented here provides insights about a number of drug delivery applications and configurations, including use of multiple needles and/or injections. There is a trade-off between maintaining low infusion volume to minimize fluid leakage away from the injection site, and increasing infusion volume to maximize lesion coverage, using the smallest number of injections possible. For a given concentration of EC-ethanol, infusion pressure increases with infusion volume. When infusion pressure exceeds a critical pressure in tissue, fluid will leak outside the depot into cracks that form in the tissue^41,42^. Thus, staying below the critical pressure by keeping infusion volumes low, is less likely to lead to crack formation and leakage away from the injection depot^35^. Counter to this, it is desirable to achieve as high a distribution volume as possible with each injection to reduce the number of injections needed. Therefore, our goal was to maximize depot volume with each injection, while also minimizing leakage outside the depot; this trade-off was determined experimentally. Infusion volume was varied in excised swine cervices and ethanol distribution was imaged with CT. CT was selected for studying the impact of various infusion volumes on distribution volume because it does not require sectioning through tissues and therefore can be used to image and analyze 3-dimensional volumes in intact samples. Further, adding a contrast agent to the injections was not necessary, since ethanol is less radio dense than tissue, and the distribution volume of ethanol could be easily segmented and quantified through applying a threshold of 0 HUs^43^. Quantification of ethanol distribution indicated that infusion volumes ≤ 500 µL led to ~ 70% of fluid retained in the tissue. Conversely, an infusion volume of 1000 µL led to significant leakage—only 20% of injected ethanol was retained. An infusion volume of 500 µL achieved an actual infusion volume of 349 µL with diminished leakage in ex vivo swine cervices. When 500 µL was injected in vivo, it led to a necrotic volume of 203.2 µL 24 h after ablation. Thus, in the total ethanol distribution volume was greater than the resulting necrotic volume. Several factors might have contributed to this, including: vascular clearance in vivo; diffusion of ethanol out from the depot; and small leakage of the depot into the vaginal canal (observed here). While insights can be gained from ex vivo experiments, extension to in vivo models is clearly mandated to fully understand this injection/ablation methodology, and to translate it to human application.

This study had limited sample sizes, and a single pilot experiment for in vivo evaluation of the hand-held injector. Targets for larger, follow up studies include: assessment of safety and necrotic volume caused by different volumes of EC-ethanol; comparison of necrotic volumes with those from current therapies for cervical dysplasia (LEEP, cryotherapy, and/or thermocoagulation); and increased overall depot volume via multiple injections or injections through a needle with multiple holes (to induce necrosis throughout the transformation zone). While this study focused on optimizing shallow injections to treat cervical dysplasia, it is of interest to extend this methodology to treat micro-invasive disease, requiring deeper injections. Notably, those could benefit from the likelihood that deeper needle insertion would likely increase the compressive stress between the needle and tissue interface, further reducing injectate loss from backflow.

In conclusion, key delivery parameters were selected to minimize fluid leakage of EC-ethanol injections in the swine cervix. Specifically, backflow was minimized through optimization of needle insertion; off-target leakage was minimized by orienting the needle bevel outwards; and crack formation was minimized by keeping infusion volumes low. These experiments guided the design of a hand-held injection device that was used to see and ablate the swine cervix in vivo*.* Overall, promising results were obtained. Similar experimental methodologies could be applied toward optimizing infusion volume for other needle-based drug delivery applications, such as intra-tumoral delivery of immunotherapies. Critical next steps in translating this method will be increased sample size for in vivo swine experiments, and comparison of ablation by EC-ethanol compared to other ablative therapies currently used to treat cervical dysplasia.

## Methods

### Experiments with porcine tissue ex vivo

Freshly excised and rinsed swine lower reproductive tracts (vagina, cervix, uterus) were obtained from Hampshire or Duroc cross-bred pigs aged 4–6 months (from Hatley farms in Hurdle Mills, North Carolina, USA). After excision, tissue was stored in Krebs–Ringer Bicarbonate Buffer (Sigma Aldrich, K4002, St. Louis, MO) on ice to preserve viability and prevent dehydration during transport to the laboratory, where experiments were conducted the same day as tissue collection. The swine cervix was located within the tract through palpitating for the interdigitating pads, which were present within the cervix but were not present within vaginal tissue. The portion of tissue below the interdigitating pads (i.e. the vagina, complete to the introitus) was removed with scissors. Additionally, the uterine fundus and horns, which were clearly identified by their shape, were removed with scissors. The cervix was then sectioned transversely into approximately 4 equal pieces, 10–15 mm in length, and stored in Krebs–Ringer Bicarbonate Buffer until experiments were conducted. Approximately 5 min after each experiment was completed, tissue was embedded in optimum cutting temperature (OCT) gel (Sakura Finetek, Torrance, CA) and flash frozen via submersion in 2-methylbutane (Sigma, Item: 277258, St. Louis, MO) in a − 80 °C freezer for 10 min. Then the tissue was wrapped in aluminum foil and stored in a − 80 °C freezer.

### Ex vivo injection methodology

**Figure 7:**
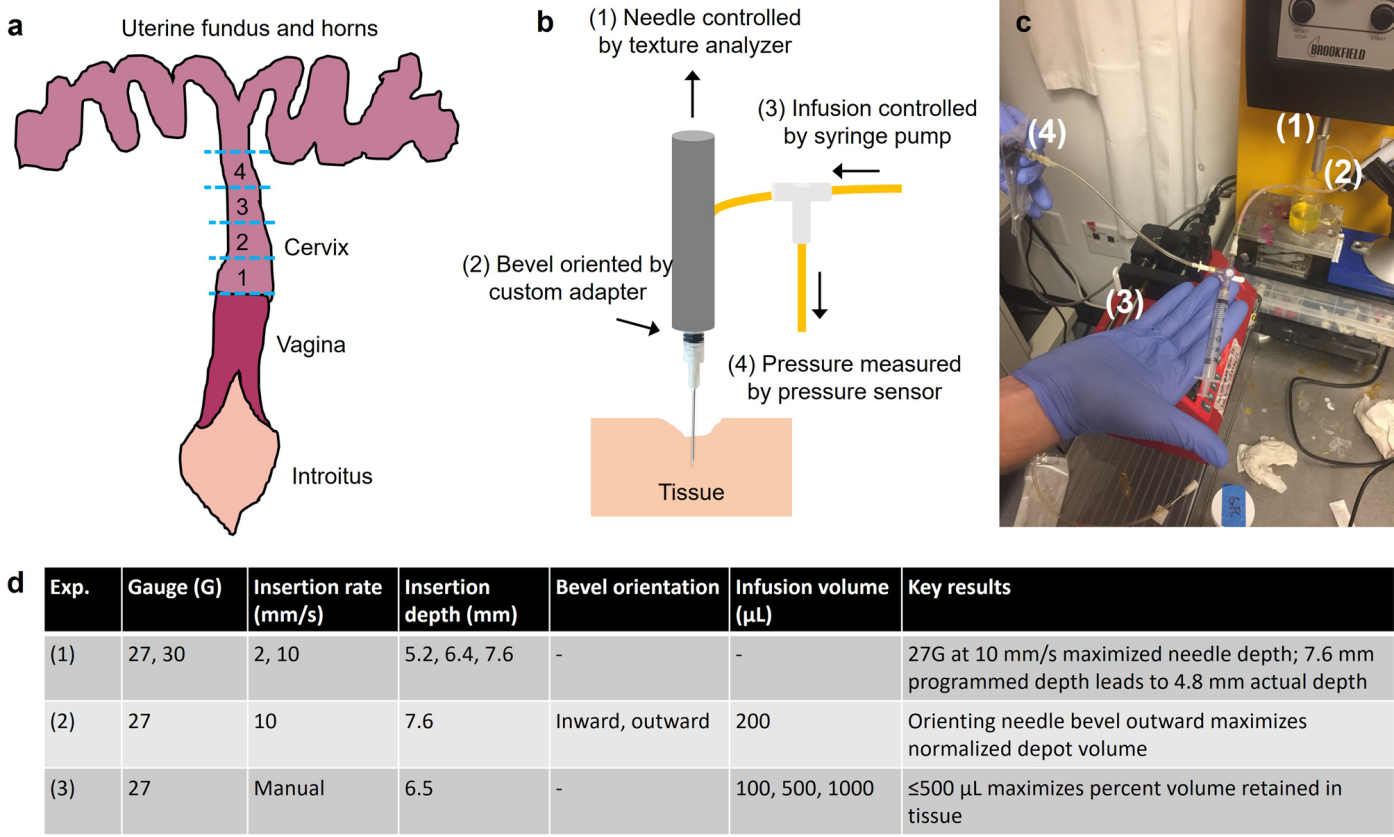
Ex vivo swine cervix experimental methodology. (**a**) The swine cervix was located within the excised reproductive tract and then sectioned transversely into approximately 4 equal pieces for experiments. (**b**) Schematic and (**c**) photograph of benchtop system, which contains (1) needle attached via a custom adapter to the Texture Analyzer to control needle insertion rate and depth. (2) The orientation of the needle bevel can be controlled through how it is attached to the custom adapter. The needle is also connected to a 3-way Luer Lok valve via tubing. The other openings from the Luer Lok are connected to (3) syringe loaded into a syringe pump to control infusion rate and volume and (4) pressure sensor to monitor pressure throughout the injection. (**d**) Summary of ex vivo swine cervix experiments with the benchtop system and key parameters that were varied in each experiment.

EC-ethanol was injected with a bench top system previously described in^35^ for ex vivo swine cervix experiments (Fig. 7). Briefly, the bench top system included a syringe pump (Item: NE-1000, New Era, Farmingdale, NY) to control infusion rate and volume, Texture Analyzer (Item: CT-3, Brookfield Ametek, Middleboro, MA) to control needle insertion rate and depth, and a pressure sensor (Item: PX26030, Omega, Bridgeport, NJ) to monitor infusion pressure. A 3-way Luer Lok valve (Cole Palmer, Vernon Hills, IL) was connected to the syringe. The other openings were connected to rubber tubing (1/4″ inner diameter, McMaster Carr, Douglasville, GA) leading to the pressure sensor and to the needle, which was attached via a custom adapter to the Texture Analyzer.

The benchtop system was used to evaluate various delivery parameters in ex vivo swine cervix tissue. Delivery parameters were systematically varied through key experiments, which were summarized in Fig. 7c. In Experiment 1, needle gauge, needle insertion rate, and needle insertion depth were varied through attaching different needles to the custom adapter and programming different insertion rates and depths into the Texture Analyzer. In Experiment 2, the bevel was either oriented inwards toward the central os or outwards towards the outer edge of the swine cervix through the custom adapter. In Experiment 3, infusion volume was varied by programming in values to the syringe pump. For all experiments, infusion rate was held constant at 10 mL/h, which was the optimal rate previously determined in^30,35^.

### Needle insertion

Different needle gauges (27G and 30G) and needle insertion rates (2 and 10 mm/s) were investigated, seeking to minimize dimpling, which occurs when tissue indents around the needle tip. This leads to reduced tissue penetration depths and increased injectate backflow along the exterior of the needle. The needle was brought into contact with the top of the ex vivo swine cervix at a location adjacent to the os. A Texture Analyzer was used to control the needle insertion rate and depth. After the needle was inserted, a mark was made on the needle at the tissue surface. After the needle was removed, the distance between the mark at tissue surface and the tip of the needle was measured with calipers (with a resolution 0.01 mm). This provided a measurement of the actual needle depth achieved in tissue. Next, the needle gauge and insertion rate were held constant (27G, 10 mm/s), and programmed needle depth was varied to determine the relationship between programmed and actual needle depth in swine cervix tissue.

### EC-ethanol formulation

EC-ethanol was prepared by mixing approximately 20 mL of 200 proof ethanol (anhydrous ethanol, Koptec, King of Prussia, PA) with EC (Item: 247499, Sigma Aldrich, St. Louis, MO) in a sealed container with a magnetic stir bar at room temperature. Concentrations of either 6% or 12% EC (EC to ethanol, weight:weight) were selected because previous experiments indicated that ≥ 6% EC minimized fluid leakage^35^. For a subset of ex vivo experiments, fluorescein (Item: F2456, Sigma Aldrich, St. Louis, MO) was mixed with EC-ethanol to visualize the distribution volume of EC-ethanol in tissue. A concentration of 0.25% fluorescein (w/v) was selected because it maximized fluorescence intensity^35^. Solutions were prepared 24 to 48 h before each injection. EC-ethanol solutions were loaded into 3 mL syringe (BD Medical, Columbus NE).

### Quantification of EC-ethanol distribution

The distribution volume of EC-ethanol in ex vivo swine cervices was evaluated in two ways: (1) through injecting tissue with EC-ethanol mixed with fluorescein, flash freezing the tissue, sectioning every 500 µm, and then imaging each section with a fluorescent microscope; or (2) through injecting tissue submerged in buffer with EC-ethanol and imaging the tissue five minutes after the injection with computed tomography (CT). Fluorescein was used to visualize the impact of bevel orientation on fluid leakage at different depths, and CT was used to assess the impact of infusion volume on injectate distribution. Ethanol has endogenous CT contrast, so no contrast agent was added to the solution.

After ex vivo swine cervices were injected with EC-ethanol mixed with fluorescein, tissue was flash frozen and stored in a −80 °C freezer as described previously. Frozen cervices were sectioned with a cryostat microtome (Microm HM 505 E, Walldorf, Germany) kept at −30 °C. Specifically, 500 µm of tissue was cut away from the top surface. The block was removed and imaged with a custom wide-field fluorescence imaging device, previously described in^35^. Briefly, the imaging device was a modified version of the Pocket colposcope^16^. Blue LEDs (470 nm) and a bandpass filter (535 nm ± 43 nm FWHM) were added in front of the camera to image fluorescein. After imaging, the block was returned to the cryostat, 500 µm of tissue cut away, and imaged again. This enabled visualization of the injectate distribution at different depths throughout the ex vivo cervices. The distribution of fluorescence within each image was quantified through applying a custom image processing algorithm described in^35^. Briefly, the region containing fluorescence was first cropped to remove artifacts located at the edge of the tissue. Then, the signal at the edge of the fluorescent region was identified and used to threshold features in the image. Feature size was quantified through using the ‘regionprops’ command in Matlab (R2018b, Mathworks Inc., Natick, MA). Total distribution volume was quantified by multiplying the area in each 2D image by the depth of the slice and then summing across all slices.

CT images were full rotation (360°) with 180 projections at a 50 ms settlement time, medium magnification (pixel size, 78.81 µm), and a field of view of 8.07 × 16.11 cm, in a 512 × 512 matrix. Images were acquired at 80 kV, 500 µA and an exposure time of 300 ms. Image processing was performed in 3D Slicer^44^. The ex vivo swine cervices were placed in Krebs–Ringer Bicarbonate Buffer within vials (20 dram polystyrene containers, Fisher Scientific, Hampton, NH) that were slightly larger than the tissue sample. Pre-ablation CT images were acquired to quantify the range of Hounsfield units (HUs) seen in the tissue. Post-ablation CT images were acquired 5 min after injection. Ethanol is less radiodense than cervix tissue and appeared black in images. Analysis of pre-ablation images indicated that a majority of tissue was above 0 HUs; thus, a threshold of 0 HUs was used to segment and quantify ethanol distribution using 3D slicer.

### Design and evaluation of a custom in vivo injection device

**Figure 8:**
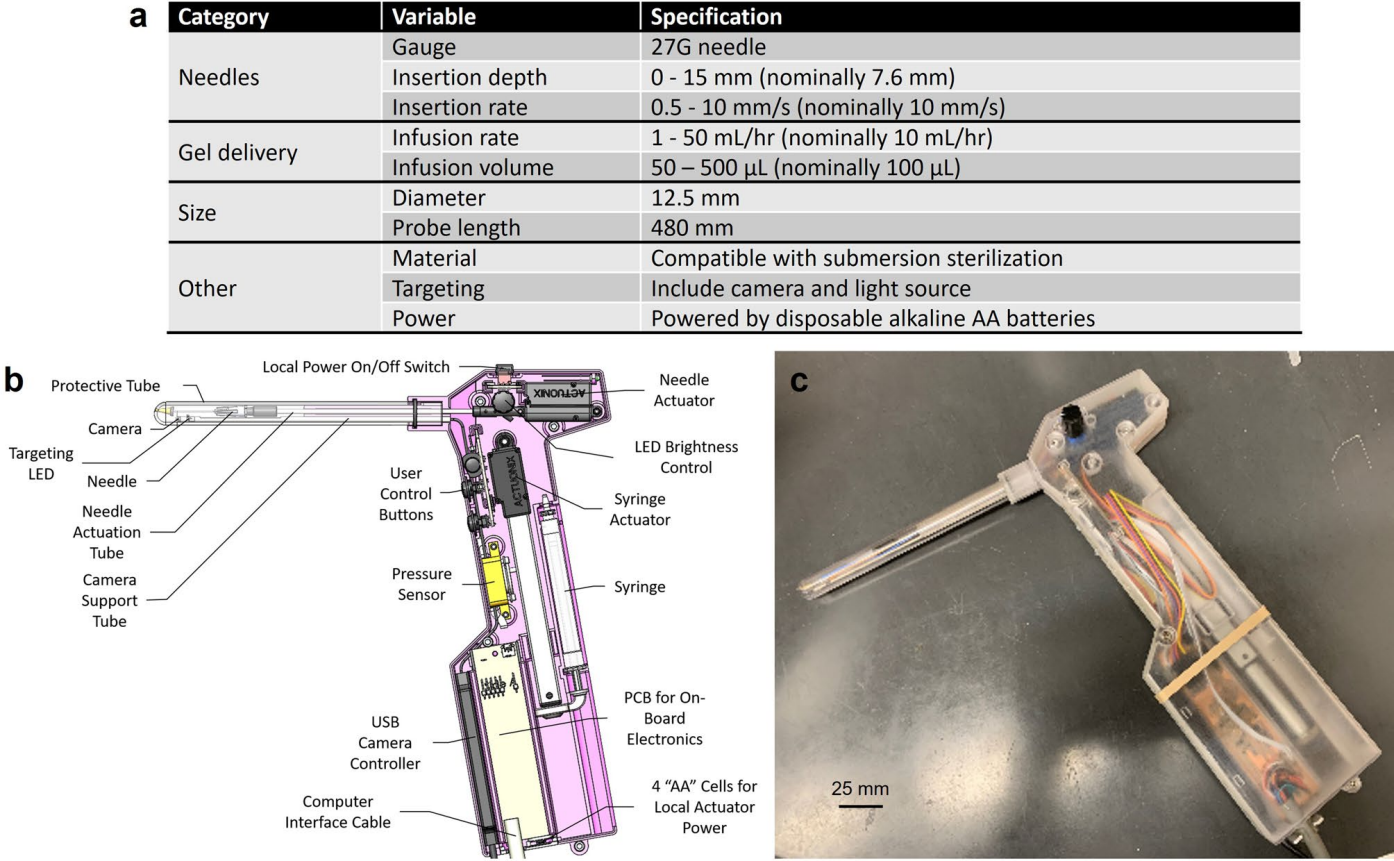
Handheld injector design. (**a**) Injector specifications to deliver EC-ethanol into the swine cervix. (**b**) Initial schematic and (**c**) prototype of automated hand-held needle injector design. Scale bar is 25 mm.

Results from ex vivo swine cervix experiments were used to guide the design of a hand-held injector that could be used to administer EC-ethanol to the swine cervix in vivo (Fig. 8). The development of an injector enabled in vivo experiments to assess both safety and efficacy. In particular, an injector was needed that could be readily inserted through the vaginal canal of a female Yorkshire pig (weight range 60–120 lbs) to the depth of the cervix, and that could control injection parameters (needle insertion rate and depth, volumetric injection rate and time). Based on anatomical considerations, design specifications included a probe diameter of 12.5 mm or less and a probe length of 480 mm. A camera (Item: MD-T1002-120X, Misumi Electronics Corporation, Schaumburg, IL) and LED light source (Item: LTW-K140SZR40, LiteOn, Taipei, Taiwan) were incorporated at the tip of the probe, to enable the user to locate the cervix in vivo, select the local region to inject, and monitor the injection. To control injection parameters, two linear actuators were incorporated into the design—one to control the needle insertion rate and depth (Item: PQ12-63-6-P, Actuonix Motion Devices, Saanichton, Canada), and the other to control the infusion rate and volume (Item: L12-100-210-6-P, Actuonix Motion Devices, Saanichton, Canada). The probe housing included a custom probe handle (material: MED-610 biocompatible resin used with Stratasys Polyjet 3D printers) to enclose the electronics and batteries, and a probe cover that slid onto the top of the handle and was secured in place via a friction fit. The probe cover was easily removed and submerged in Cidex OPA for disinfection between uses. The probe cover also contained a funnel at the probe tip to catch the needle, which accounted for slight variations in manufacturing (up to 3° in any direction off the axis of the plastic part of the needle). Lastly, a pressure sensor (Item: 86A, TE Connectivity, Schaffhausen, Switzerland) was connected in series between the syringe and needle to monitor infusion pressure during injections. Needle insertion rate and depth and infusion rate and volume could be programmed by a custom LabVIEW (National Instruments, Austin, TX) interface, through which a video of the injection and the infusion pressure could also be recorded.

To evaluate the injector’s performance, a series of bench tests was performed. Insertion depth was determined by measuring the distance from the tip of needle to the probe housing with calipers. A video of the insertion was recorded with a USB webcam mounted perpendicular to the axis of motion, from which the insertion rate was verified through quantifying distance the needle moved over time. Specifically, time was calculated by determining the number of frames between the initial to final distance, and then converting that difference to seconds by dividing by the frame rate of the video. Infusion volume was determined by infusing a programmed volume and weighing its mass, which could be converted to volume. The infusion rate was verified by measuring the time to reach the programmed volume. Lastly, the infusion pressures associated with each injection were recorded with the manometer.

### Pilot in vivo study

The animal study protocol was approved by the Duke University Institutional Animal Care and Use Committee (protocol number A208-18-08) and was performed in accordance with relevant guidelines and regulations and in compliance with the ARRIVE guidelines. The procedure was performed under isoflurane anesthesia. A female Yorkshire swine weighing 92.5 lbs (42 kg) was obtained from Wesley Hooper Farms and housed for one week prior to initiation of experiment procedures. After initiation, the swine was anesthetized with isoflurane and injected with 500 µL EC-ethanol, which is far below the lowest published lethal dose for a mammal of 1600 mg/kg (approximately 85 mL for a 42 kg pig). Vital signs, including temperature, heart rate, and blood oxygen were recorded throughout the procedure. Mobility and respiratory distress were documented every 15 min during and after the procedure, for approximately 2 h. Mobility was quantified by measuring the time to walking after the pig recovered from anesthesia. Respiration was visually observed. Approximately 24 h after treatment, the pig was euthanized, and the cervix was excised and sectioned into 4 pieces that were 10–15 mm in length. The tissue was embedded in OCT, flash frozen, and stored in a −80 °C freezer as described above.

### Processing for pathology assessment

Tissue containing necrotic regions was sectioned with a cryostat microtome kept at −30 °C. Specifically, 10 µm sections were cut every 500 µm and mounted onto glass slides. Sections were stained with NADH-diaphrose, which stained viable cells blue and did not stain necrotic regions^45^. Stained slides were imaged with an inverted bright-field microscope with a 5X objective (Item: DMi8, Leica Microsystems, Buffalo Grove, IL). Necrotic regions were identified in each image and outlined using the ‘imfreehand’ command within Matlab to create a binary mask from which the area of the outlined region was calculated. Necrotic distribution volume was quantified by multiplying the area in each 2D image by the depth of the slice and then summing across all slices.

### Statistical analysis

Wilcoxon rank sums (non-parametric, two-tailed, alpha = 0.95) were used to determine whether injection parameters led to significant differences in measured output variables, such as actual needle depth or normalized depot volume. A significance level of p = 0.05 was considered to reject the null hypothesis for all analyses.

## Data Availability

The datasets generated during and analyzed during the current study are available from the corresponding author on reasonable request.
